# ICTV Virus Taxonomy Profile: Bromoviridae 2025

**DOI:** 10.1099/jgv.0.002069

**Published:** 2025-01-31

**Authors:** Jeremy R. Thompson, Tomás Canto, John P. Carr, Vicente Pallás, Dana Šafářová

**Affiliations:** 1 Plant Health and Environment Laboratory, 231 Morrin Road, St Johns, Auckland, 1072, New Zealand; 2 Margarita Salas Center for Biological Research (CIB-CSIC), Spanish Council for Scientific Research (CSIC), Ramiro de Maeztu 9, 28040 Madrid, Spain; 3 Department of Plant Sciences, University of Cambridge, Cambridge CB2 3EA, UK; 4 Institute for Plant Molecular and Cell Biology, Polytechnic University of Valencia-CSIC, Avenida de los Naranjos s/n, 46022 València, Spain; 5 Department of Cell Biology and Genetics, Faculty of Science, Palacký University, Olomouc, Šlechtitelů 27, 783 71 Olomouc-Holice, Czech Republic

**Keywords:** *Bromoviridae*, ICTV Report, taxonomy

## Abstract

*Bromoviridae* is a family of plant viruses with tripartite, positive-sense RNA genomes of about 8 kb in total. Genomic RNAs are packaged in separate virions that may also contain sub-genomic, defective or satellite RNAs. Virions are variable in morphology (spherical or bacilliform) and may be transmitted between hosts mechanically, via pollen, or non-persistently by insect vectors. Members of the family are responsible for major disease epidemics in fruit, vegetable and fodder crops such as tomatoes, cucurbits, bananas, fruit trees, common beans and alfalfa. Since the adoption of metagenomic high-throughput sequencing methodologies, there has been a notable increase in the number of species in the genus *Ilarvirus*. This is a summary of the International Committee on Taxonomy of Viruses (ICTV) Report on the family *Bromoviridae,* which is available at ictv.global/report/bromoviridae.

## Virion

**Fig. 1.**
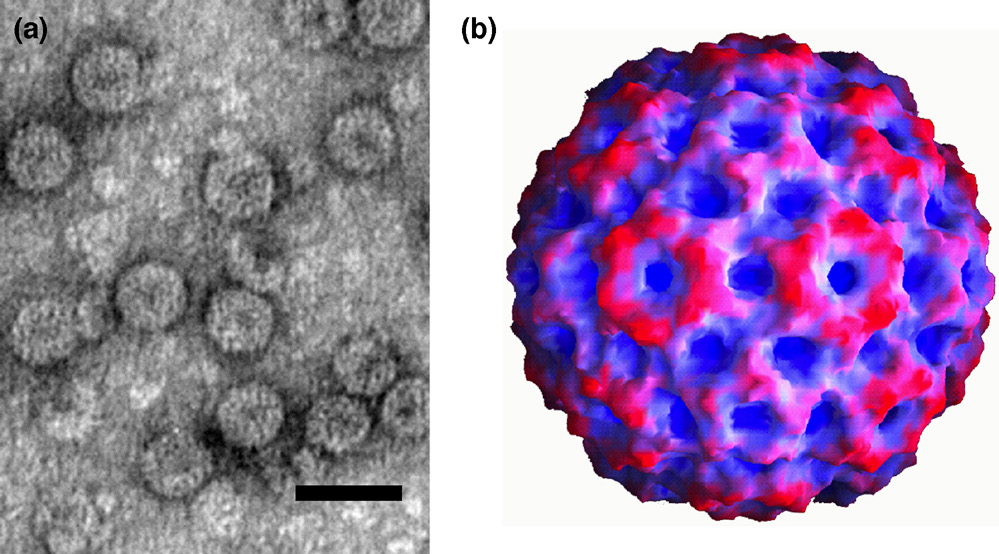
Particles of cucumber mosaic virus. (**a**) Negative-contrast electron micrograph (bar 50 nm, courtesy of A. De Stradis, IPSP-CNR, Bari, Italy) and (**b**) reconstruction (courtesy of Dr K.L. Perry, Cornell University, Ithaca, New York, USA, Dr T. Smith, University of Texas, Galveston, Texas, USA, and A. Paredes, NCTR/ORA, Arkansas, USA).

Virions are either spherical or quasi-spherical (Table 1 and Fig. 1), having *T*=3 icosahedral symmetry and a diameter of 26–35 nm (genera *Anulavirus*, *Bromovirus*, *Cucumovirus* and *Ilarvirus*), or bacilliform (genera *Alfamovirus*, *Ilarvirus* and *Oleavirus*) with dimensions of 18–26 nm by 30–85 nm. Genomic RNAs are packaged in separate virions that may also contain sub-genomic, defective or satellite RNAs [1].

**Table 1.** Characteristics of members of the family *Bromoviridae*

Example:	brome mosaic virus (RNA1: X02380; RNA2: X01678; RNA3: J02042), species *Bromovirus BMV*
Virion	Spherical or quasi-spherical (26–35 nm diameter) or bacilliform (18–26 nm by 30–85 nm) particles
Genome	Three segments of linear, positive-sense RNA, comprising about 8 kb in total
Replication	On cytoplasmic membranes with genomic RNAs acting as mRNAs; coat protein may be required for genome activation
Translation	Directly from genomic or sub-genomic RNA
Host range	Broad or restricted range of plants depending on the virus species
Taxonomy	Realm *Riboviria*, kingdom *Orthornavirae*, phylum *Kitrinoviricota*, class *Alsuviricetes*, order *Martellivirales*; >5 genera, including >45 species

## Genome

**Fig. 2.**
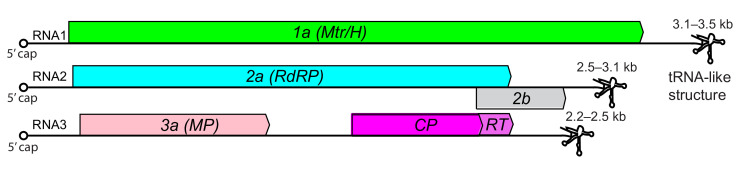
Generalized genome organization for members of the family *Bromoviridae*. Mtr/H, methyltransferase and helicase; RdRP, RNA-directed RNA polymerase; MP, movement protein; CP, coat protein. 2b protein only in some cucumoviruses and ilarviruses. Readthrough product of CP ORF (RT) only in some ilarviruses [6].

The genome of about 8 kb is split among three linear, positive-sense RNAs that have 5′-terminal cap structures (Fig. 2). The 3′-termini either form tRNA-like structures that can be aminoacylated (genera *Bromovirus* and *Cucumovirus*) or other structures that are not aminoacylated (genera *Alfamovirus*, *Anulavirus*, *Ilarvirus* and *Oleavirus*).

## Replication

Replication of genomic and sub-genomic RNAs occurs on cytoplasmic membranes via full-length negative-sense RNA synthesis and subsequent positive-sense RNA synthesis. Coat protein may be required for the activation of replication (*Alfamovirus* and *Ilarvirus*); the ratio of cytoplasmic/nuclear coat protein accumulation modulates viral gene expression (*Alfamovirus*) [1].

## Pathogenicity

Alfalfa mosaic virus (genus *Alfamovirus*) infects many herbaceous and some woody hosts, inducing systemic mottling and ‘calico’ mosaic. Pelargonium zonate spot virus (genus *Anulavirus*) infects tomato plants, which display stunting, concentric chlorotic or necrotic rings and line patterns on leaves, stems and fruit [2]. Members of the genus *Bromovirus* infect some Poaceae or Fabaceae and elderberry, inducing mosaic, brown streaks and reduced seed yield. Some strains of cucumber mosaic virus (genus *Cucumovirus*) support a 330–390 nt satellite RNA that may induce necrosis in tomato, chlorosis in tomato, tobacco and pepper or attenuate disease symptoms. Hosts include fruit crops, vegetables, ornamentals and weeds [3]. Members of the genus *Ilarvirus* infect fruit trees and some herbaceous crops. Prunus necrotic ringspot virus and prune dwarf virus cause stunting and necrotic lesions on the leaves of sweet cherry, sour cherry, plum and peach trees [4]. Olive latent virus 2 (genus *Oleavirus*) has been recorded in olive and in castor beans. Infections are asymptomatic in olive but produce a yellowish vein netting and mottling of the leaves of castor bean plants [5].

## Taxonomy

Current taxonomy: ictv.global/taxonomy. Genus assignments are based on virus host range, genome content and vector. With the exception of *Ilarvirus* and *Oleavirus*, members of a genus are monophyletic by phylogenetic analysis of the RNA-directed RNA polymerase. Virus transmission is by aphids (members of the genera *Alfamovirus* and *Cucumovirus*), thrips and/or pollen (*Anulavirus* and *Ilarvirus*), beetles (*Bromovirus*) or is unknown (*Oleavirus*). Species demarcation criteria include natural host range, mode of transmission, particle morphology and physicochemical properties, genome structure and replication strategies, an ability to support replication of defective RNAs and satellite RNAs, and <85% amino acid identity for the whole 2a protein.

## Resources

Full ICTV Report on the family *Bromoviridae*: www.ictv.global/report/bromoviridae.

## Abbreviations

ICTV: International Committee on Taxonomy of Viruses
RdRP: RNA-directed RNA polymerase
